# A randomised clinical study investigating efficacy of a stannous fluoride toothpaste in improving gingival health after 3 weeks’ use

**DOI:** 10.1186/s12903-021-01727-5

**Published:** 2021-09-12

**Authors:** Amina Acherkouk, Nisha Patel, Andrew Butler, Pejmon Amini

**Affiliations:** 1 GSK Consumer Healthcare, St George’s Avenue, Weybridge, Surrey, KT13 ODE UK; 2 Silverstone Research Group, 6707 West Charleston Blvd, Las Vegas, NV 89146 USA

**Keywords:** Dental plaque, Gingivitis, Periodontal diseases, Tin fluorides, Toothpastes

## Abstract

**Background:**

This examiner-blind, stratified, parallel study aimed to evaluate the anti-gingivitis efficacy of a non-aqueous (anhydrous) 0.454% w/w stannous fluoride toothpaste (‘Test’) versus a sodium monofluorophosphate toothpaste (‘Control’) in people with clinically-confirmed mild-moderate gingivitis. Plaque-induced gingivitis can progress to irreversible periodontitis if left untreated. This can be controlled by an effective oral hygiene regimen such as one including toothbrushing with a toothpaste containing the chemotherapeutic agent stannous fluoride. Long-term studies over 4–12 weeks have shown the efficacy of stannous fluoride; however, shorter term studies are needed to examine if the effects on measures of gingivitis and plaque control occur sooner.

**Methods:**

Eligible participants were randomised to 3 weeks’ twice-daily brushing (for 1 min) with Test or Control toothpastes. The primary efficacy variable was between-treatment difference in Bleeding Index (BI) at 3 weeks; secondary variables were between-treatment differences in number of bleeding sites, modified Gingival Index (MGI), and Turesky modification of the Quigley–Hein Plaque Index (TPI) at Weeks 2 and 3.

**Results:**

A statistically significant (*p* < 0.0001) lower BI score was reported for Test (n = 65) versus Control (n = 65) groups at Week 2 (mean difference: − 0.07 [95% CI − 0.9, − 0.05]; 32.7% difference) and Week 3 (mean difference: − 0.06 [95% CI − 0.8, − 0.04]; 29.2% difference). The Test group also demonstrated statistically significant lower (all *p* < 0.0001 versus Control) number of bleeding sites (Weeks 2/3 mean difference [95% CI]: − 10.04 [− 12.3, − 7.5]/ − 8.2 [− 11.1, − 5.3] sites; 33.0%/29.3% difference); MGI score (Weeks 2/3 mean difference [95% CI]: − 0.09 [− 0.13, − 0.06]/ − 0.10 [− 0.14, − 0.06]; 4.3%/4.7% difference); overall TPI score (Weeks 2/3 mean difference [95% CI]: − 0.45 [− 0.55, − 0.35/ − 0.42 [− 0.53, − 0.30] difference; 16.0%/15.1% difference) and interproximal TPI score (Weeks 2/3 mean difference [95% CI]: − 0.42 [− 0.52, − 0.30]/ − 0.41 [− 0.52, − 0.29]; 14.6%/14.1% difference). Both toothpastes were generally well tolerated.

**Conclusion:**

Three weeks’ twice-daily brushing with the 0.454% w/w stannous fluoride Test toothpaste compared to the Control toothpaste led to statistically significant lower gingival bleeding, gingival inflammation and plaque levels in adults with mild-moderate gingivitis. These results indicate that plaque and gingivitis-reducing benefits of 0.454% w/w stannous fluoride may be seen from 2 weeks’ use.

*Trial registration* ClinicalTrials.gov Identifier: NCT04050722; 08/08/2019.

## Background

While gingivitis, an inflammatory response to dental plaque [1], is a reversible condition, it can progress to irreversible periodontitis if left untreated, eventually leading to tissue, tooth and bone loss [2]. The maintenance of gingival health is therefore paramount as, currently, periodontitis is reported to affect 5% to 20% of the world’s population [3].

Gingivitis can be prevented and resolved through effective plaque control, primarily via the mechanical removal of plaque by toothbrushing. Where adequate plaque control cannot be achieved by such means, this can be augmented with the use of antimicrobial agents in oral healthcare products [4–6]. Accordingly, antimicrobial agents such as metal salts, cetylpyridinium chloride and chlorhexidine have been included in daily use toothpastes and mouth rinse formulations for many years [4, 7]. These complement mechanical plaque removal by inhibiting the growth of bacteria (via bacteriostatic and/or bactericidal activity) in the areas of dentition less accessible to the toothbrush and by interfering with re-colonisation of plaque bacteria [8].

In this study, a toothpaste containing the antimicrobial agent stannous fluoride (SnF_2_) was investigated due to the antimicrobial properties of stannous ions (Sn [II]), which have been shown to reduce bacterial biomass/virulence and inhibit bacterial metabolism [9–11]. This action is supported by numerous clinical studies where the anti-gingivitis/anti-plaque efficacy of 0.4% to 0.454% w/w SnF_2_ toothpastes has been shown [7, 12], including studies where an anhydrous formulation, which aids SnF_2_ stability within the toothpaste, is used [13–15]. These previous studies have investigated the efficacy of the 0.454% w/w SnF_2_ toothpaste at time-points ranging from 4 to 24 weeks [13–15]; however, it is of interest to understand if this efficacy can be seen at earlier timepoints.

This study evaluated the anti-gingivitis efficacy, following dental prophylaxis, of an anhydrous 0.454% w/w SnF_2_ toothpaste after 2 and 3 weeks twice-daily brushing for 1 timed minute. Efficacy was compared to a negative control toothpaste containing sodium monofluorophosphate. The primary objective was evaluation of clinical efficacy as measured by the Bleeding Index (BI) [16] after 3 weeks’ use. Secondary objectives included BI at 2 weeks and number of bleeding sites, modified Gingival Index (MGI) [17] and Turesky Modification of the Quigley Hein Plaque Index scores (TPI) [18, 19] after 2 and 3 weeks’ use.

## Methods

This was a 3 week, randomised, stratified, examiner-blind, two-treatment, parallel-group clinical trial performed at a clinical study centre in Las Vegas, USA. The study was carried out in full compliance with the International Council for Harmonisation of Technical Requirements for Registration of Pharmaceuticals for Human Use, all applicable local good clinical practice regulations and participant privacy requirements and the ethical principles outlined in the Declaration of Helsinki. The final study protocol, informed consent forms and all other information that required pre-approval were reviewed and approved by an independent institutional review board (IntegReview IRB, Austin, Texas, USA. IRB number: IRB00003657/IORG0000689). Anonymised individual participant data and study documents can be requested for further research from www.clinicalstudydatarequest.com. ClinicalTrials.gov Identifier: NCT04050722; registration date 08/08/2019.

### Participants

Eligible participants provided written informed consent before study procedures commenced. Eligibility included having mild-moderate plaque-induced gingivitis, as confirmed by visual examination by a single investigator, and being aged 18 to 65 years and in good health with no significant/relevant medical or oral/dental conditions. Participants needed to have at least 20 natural teeth, with ≥ 40 surfaces gradable by the clinical indices used in the study. Excluded teeth included third molars and any with gross caries, calculus, orthodontic bands/bonds, crowns or extensive restoration. At baseline examination, eligibility included a mean whole mouth MGI score of between 1.75–2.30, a mean overall TPI score of ≥ 1.5 and ≥ 20 bleeding sites.

Participants with any medical condition or those who were taking any medications that could affect gingival condition/bleeding were excluded. This included those with current or within 14 d of baseline use of any antibacterial mouthwash or oral care product, antibiotics, anti-inflammatory or anti-coagulant medications, or any other systemic medication. Additional medical exclusion criteria included: current smoker, or who had stopped within 6 months of screening; current user of any smokeless form of tobacco; pregnancy; breast feeding; tongue/lip piercing; intolerance/hypersensitivity to study materials or participation in another clinical trial within 30 d of screening. Dental exclusion criteria included: periodontitis and/or periodontal treatment within 12 m of screening; partial/full dentures and teeth whitening treatment or dental prophylaxis within 12 w of screening.

### Study visits and clinical procedures

At screening (Visit 1), participants’ demographics and medical history were recorded, followed by an oral examination including gingival assessment. Eligible participants were instructed to continue brushing using their usual toothpaste and routine until the baseline visit (Visit 2), which occurred 1–28 d after Visit 1. On the evening before the baseline visit, participants abstained from oral hygiene procedures, allowing for 10–18 h plaque accumulation prior to their study centre visit. At Visit 2, a single dental examiner performed a full oral soft tissue (OST) examination, then assessments of gingival inflammation (MGI), gingival bleeding (BI), and supra-gingival plaque (TPI) were conducted.

For the MGI [17], assessment was carried out for each scorable tooth at the gingival margin and interdental papillae of the lingual and buccal surfaces. Each surface was scored on a 5-point scale from 0 (absence of inflammation) to 4 (severe inflammation) with MGI calculated as the mean score of all scored tooth sites.

For each scorable tooth, bleeding was assessed on both lingual and facial gingival surfaces. Three scores (distal, body, mesial sites) were recorded lingually/palatally and three buccally/labially. Bleeding was scored as 0 (no bleeding after 30 s), 1 (bleeding on probing after 30 s) or 2 (immediate bleeding on probing) [16] with a site considered as ‘bleeding’ if the BI score was 1 or 2. For each participant, overall BI score was calculated by adding up the number of all individual bleeding sites scored and dividing by the number of sites assessed.

Plaque was disclosed according to manufacturer’s instructions (Trace^®^ Solution; Young Dental, Earth City, MO, USA) then was assessed using the TPI [19] on mesial, distal and mid sites of lingual and buccal surfaces of each gradable tooth. Plaque presence was scored on a 6-point scale from 0 (no plaque) to 5 (plaque covering two-thirds or more of tooth crown). Overall TPI and interproximal TPI scores were calculated respectively as mean TPI over all tooth sites and mean TPI over interproximal sites (distal and mesial).

Once baseline assessments had been carried out, a dental prophylaxis, using a conventional prophylaxis paste followed by flossing, was carried out by a clinical staff member. This was checked by a second examiner who confirmed all plaque had been removed by use of disclosing solution. If found, any remaining plaque was removed by dental polishing with the prophylaxis paste to bring the participant’s TPI score to 0.

Randomisation was undertaken by the Biostatistics Department of the study sponsor using validated internal software. Participants were stratified according to baseline MGI score (low: ≤ 2.00; high: > 2.00) and sex (female/male). They were randomised to use either the ‘Test’ toothpaste containing 0.454% w/w SnF_2_ (1100 ppm fluoride; Sensodyne Repair and Protect; GSK Consumer Healthcare, Brentford, UK; USA Marketplace product) or the ‘Control’ toothpaste containing 1100 ppm fluoride as sodium monofluorophosphate (Colgate^®^ Cavity Protection, Colgate-Palmolive Co; New York, USA; USA Marketplace product). Toothpaste tubes were overwrapped in white vinyl to aid study blinding. The examiner, study statistician, and sponsor employees were blinded to treatment allocation. The examiner was not permitted in the room where study products were dispensed or stored.

Participants were supplied with an Aquafresh^®^ Clean Control toothbrush (GSK Consumer Healthcare, Brentford, UK; USA Marketplace product) for use throughout the study. They were instructed to apply a full strip of their assigned toothpaste to cover the head of the toothbrush and brush their teeth twice daily (morning and evening) for one timed minute, as this is typically the time most people spend brushing their teeth [20]. Written toothbrushing instructions were provided and first use of study products was carried out under supervision at the study centre. Participants recorded each use of study product in a provided diary. They abstained from use of all other oral hygiene products from the baseline visit.

Participants returned to the study site at Weeks 2 (± 2 d) and 3 (± 2 d) (Visits 3 and 4, respectively). They presented with overnight plaque, having abstained from toothbrushing the previous night and underwent a full OST examination followed by MGI, BI and TPI assessments. Diary entries were assessed to ensure study product use compliance.

### Safety

Adverse events (AEs) and incidents were recorded from first use of study products until 5 d following last use of the study product. Clinical judgment was used to assess the relationship of any AE to the study product by the examiner.

### Statistical analysis

This study was to be considered successful if a statistically significant difference between the adjusted mean BI scores of the two study product groups at Week 3 was observed in favour of the Test toothpaste. A sufficient number of participants were to be screened to randomise approximately 130 (65 per group), to ensure 60 evaluable participants per group completed the study. With this number per group, it was estimated to detect a 0.1 difference in BI (standard deviation [SD] = 0.17) [14] with 90% power and a 2-sided 5% significance level. This would represent approximately a 23% difference between study product groups. This estimate allowed detection of a 5% difference in a key secondary variable, MGI, with 90% power. All statistical analyses were performed using SAS Studio version 9.4 (SAS Institute Inc, Cary, NC, USA).

The efficacy analysis was performed on a modified intent-to-treat (mITT) population, defined as all randomised participants who received at least one dose of study treatment and had at least one post-baseline efficacy measurement. Safety analysis was performed on the safety population, defined as all randomised participants who received at least one dose of study treatment.

The primary efficacy endpoint was mean BI score at Week 3. Secondary endpoints were BI score at Week 2 and, at Weeks 2 and 3, number of bleeding sites, MGI overall, TPI overall and TPI interproximal. For all endpoints, statistical testing was conducted at an unadjusted two-sided significance level of 0.05. Statistical analyses for all measures were performed using analysis of covariance (ANCOVA), including treatment group, sex and (except for MGI), baseline MGI stratification as factors and the respective baseline clinical index score as a covariate (e.g., mean baseline BI score for BI analysis). Adjusted means of the two treatments and treatment difference were provided together with 95% confidence intervals (CIs) and *p*-values. The assumption of normality and homogeneity of variance was investigated and considered satisfied. Percent difference was calculated as: 100*(adjusted mean difference/adjusted mean of negative control toothpaste).

### Intra-examiner repeatability

To assess intra-examiner repeatability, repeat TPI and MGI assessments were carried out by a single examiner on 30 participants at baseline and Week 3 visits. Repeat examinations were performed at least 10 min apart and, where possible, were separated by assessment of another participant. Replicate BI examinations could not be carried out due to the influence of the first assessment on the repeat examination. Assessments on each tooth at each visit were cross-tabulated and a weighted Kappa coefficient (κ) was calculated, along with the 95% CI. Repeatability was compared against pre-defined values as excellent if κ > 0.75; fair to good if 0.4 ≤  κ  ≥ 0.75; and poor if κ  < 0.4.

## Results

**Fig. 1:**
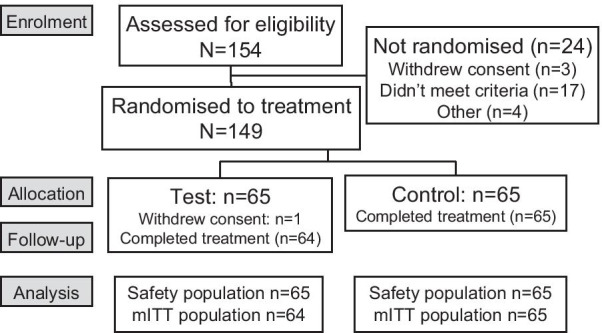
Study flow. *mITT* modified intent to treat

The first participant was enrolled on October 7th, 2019; the final participant completed the study on November 13th, 2019. Of 154 screened participants, 130 were randomised to treatment (65 per group) and were included in the mITT and safety populations; 129 participants completed the study (Fig. 1). Participants were aged between 18 and 65 yrs (mean 39.4 yrs; SD: 11.95), the majority were female (60.8%) and all but eight participants had an MGI score > 2.00 at baseline. Baseline characteristics and strata were well balanced between treatment groups (Table 1).

**Table 1:** Baseline demographics and characteristics (Safety population)

	Test (n = 65)	Control (n = 65)
Sex, n (%)		
Female	39 (60.0%)	40 (61.5%)
Male	26 (30.0%)	25 (29.2%)
Heritage, n (%)		
White: Caucasian/European/Arabic/North African	39 (60.0%)	30 (46.2%)
African American/African	15 (23.1%)	19 (29.2%)
Asian: Central/South/East/South East/Japanese	4 (6.2%)	6 (9.2%)
Native Hawaiian/Pacific Islander	1 (1.5%)	3 (4.6%)
Multiple	6 (9.2%)	5 (7.7%)
Mean age, years (SD)	39.2 (11.50)	39.4 (11.95)
Range, years	18–65	18–64
Stratification, n (%)		
Male, baseline MGI ≤ 2.00	1 (1.5%)	2 (3.1%)
Male, baseline MGI > 2.00	25 (38.5%)	23 (35.4%)
Female, baseline MGI ≤ 2.00	3 (4.6%)	2 (3.1%)
Female, baseline MGI > 2.00	36 (55.4%)	38 (58.5%)

The Kappa scores for MGI and TPI were 0.83 (95% CI 0.81, 0.85) and 0.90 (95% CI 0.88, 0.91), respectively, thereby demonstrating excellent repeatability.

### Clinical outcome

**Fig. 2:**
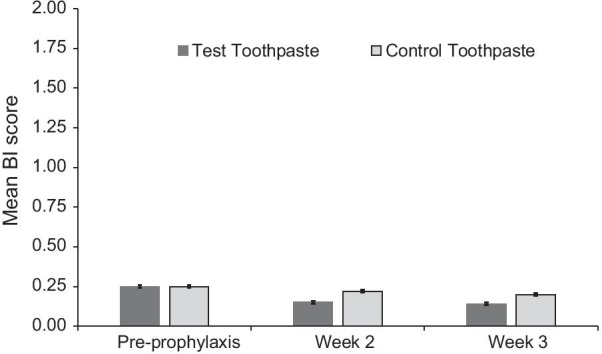
Mean Bleeding Index (BI) score ± standard error* (modified intent to treat population). *Raw mean at baseline (pre-prophylaxis); adjusted mean at Weeks 2 and 3 (post-prophylaxis following baseline assessment). BI score ranges from 0 (no bleeding at 30 s after probing) to 2 (immediate bleeding on probing)

**Fig. 3:**
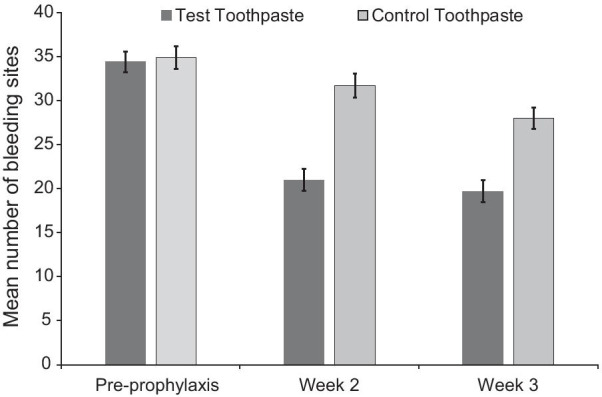
Mean number of bleeding sites ± standard error* (modified intent to treat population). *Raw mean at baseline (pre-prophylaxis); adjusted mean at Weeks 6 and 12 (post-prophylaxis following baseline assessment). BI score ranges from 0 (no bleeding at 30 s after probing) to 2 (immediate bleeding on probing); a site was considered as 'bleeding' if the BI score was 1 or 2

**Fig. 4:**
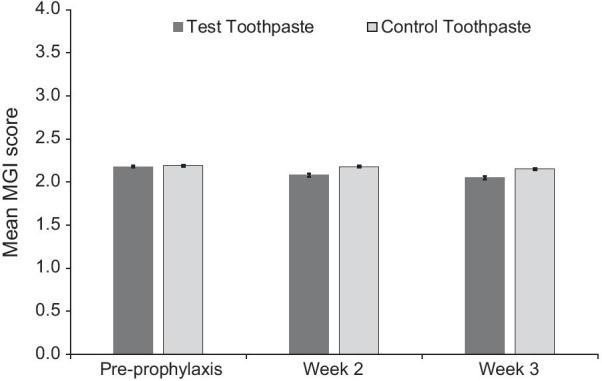
Mean modified Gingival Index (MGI) score ± standard error* (modified intent to treat population). *Raw mean at baseline (pre-prophylaxis); adjusted mean at Weeks 6 and 12 (post-prophylaxis following baseline assessment)**.** MGI score ranges from 0 (absence of inflammation) to 4 (severe inflammation)

**Fig. 5:**
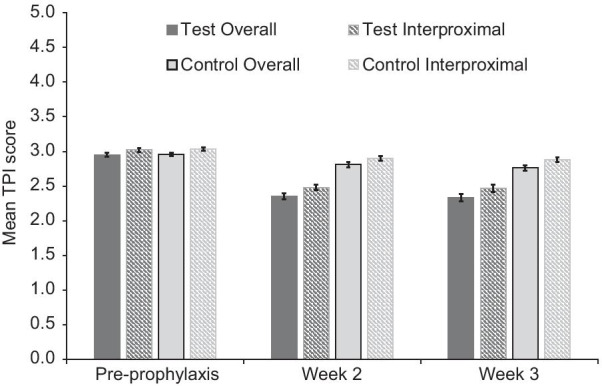
Mean overall and interproximal Turesky Plaque Index (TPI) score ± standard error* (mITT population). *Raw mean at baseline (pre-prophylaxis); adjusted mean at Weeks 6 and 12 (post-prophylaxis following baseline assessment). TPI score ranges from 0 (no plaque) to 5 (severe plaque covering two-thirds or more of the tooth crown)

Mean pre-prophylaxis scores (± standard error) for BI (Fig. 2), number of bleeding sites (Fig. 3), MGI (Fig. 4), TPI (Fig. 5) and the adjusted difference between treatment groups for all endpoints at Weeks 2 and 3 (Table 2) are shown in their respective figures or tables.

**Table 2:** Efficacy endpoints: Difference in adjusted mean scores between Test and Control groups (mITT population)

	Difference^a^ (95% CI)	*p* value	% Diff^b^
Bleeding Index score			
Week 2	− 0.07 (− 0.09, − 0.05)	< 0.0001	32.7%
Week 3	− 0.06 (− 0.08, − 0.04)	< 0.0001	29.2%
Number of bleeding sites			
Week 2	− 10.4 (− 13.3, − 7.5)	< 0.0001	33.0%
Week 3	− 8.2 (− 11.1, − 5.3)	< 0.0001	29.3%
Modified Gingival Index score			
Week 2	− 0.09 (− 0.13, − 0.06)	< 0.0001	4.3%
Week 3	− 0.10 (− 0.14, − 0.06)	< 0.0001	4.7%
Overall Turesky Plaque Index score			
Week 2	− 0.45 (− 0.55, − 0.35)	< 0.0001	16.0%
Week 3	− 0.42 (− 0.53, − 0.30)	< 0.0001	15.1%
Interproximal Turesky Plaque Index score			
Week 2	− 0.42 (− 0.52, − 0.33)	< 0.0001	14.6%
Week 3	− 0.41 (− 0.52, − 0.29)	< 0.0001	14.1%

^a^From ANCOVA analysis; difference is Test toothpaste minus Control toothpaste; a negative difference favours the Test toothpaste

^b^Percentage difference between group scores calculated as (treatment difference/adjusted mean of reference treatment)*100%

The primary objective was met as at Week 3 there was a statistically significant lower BI score in the Test group compared with the Control group (*p* < 0.0001 for both). A similar significant difference was shown at Week 2 (*p* < 0.0001) with similar percentage differences at both weeks, showing sustained BI score differences (Table 2).

At Weeks 2 and 3, the Test group also demonstrated statistically significant lower number of bleeding sites, MGI score, total TPI score and interproximal TPI score (all *p* < 0.0001) compared to the Control group. For all measures, percentage differences were similar between the weeks, showing sustained gingival differences and plaque control (Table 2).

### Safety

There were no serious AEs and no withdrawals due to treatment-emergent AEs (TEAEs). Four participants (6.2%) in the Test toothpaste group reported six TEAEs (one episode each of dry mouth and gingival injury; one participant with two episodes of angular cheilitis; two episodes of traumatic ulcer in two participants). Three participants (4.6%) in the Control toothpaste group reported five TEAEs (one episode each for dry mouth and gingival injury; three episodes in one participant of gingival ulceration). All TEAEs were mild in intensity and were resolved by study completion. One TEAE was considered treatment related: dry mouth, occurring once in the Test toothpaste group, once in the Control group.

## Discussion

The results of this study indicate a difference in adjusted mean scores between the Test and Control groups with regard to BI score, number of bleeding sites, MGI score and overall and interproximal TPI scores. This was shown at both Weeks 2 and 3, all in favour of the Test group.

Examination of gingival bleeding on probing as an indication of gingival inflammation is important as maintenance of periodontal and gingival health can be predicted by a low number of bleeding sites [21]. The effects of SnF_2_ containing toothpastes on gingivitis have been described in several other clinical studies and reviews [22, 23]; however, many of these were carried out over long time periods such as 12 weeks [14, 24] or 6 months [7, 12, 25, 26].

It has long been reported that recordable gingival health benefits of chemotherapeutic containing toothpastes may be feasible after 2 to 3 weeks of use [27, 28]. This is confirmed in this current study with results showing significant differences in BI and number of bleeding sites between the SnF_2_ toothpaste and the Control at both 2 and 3 weeks. These findings are consistent with prior studies with a similar SnF_2_ toothpaste over longer time periods [13–15]. Additionally, interim results of a 12 week study of a similar toothpaste showed that on examination at Week 4, there were also significant differences, and similar percentage changes, in BI (33.4%) and number of bleeding sites (33.5%) to the findings of this current study [14]. Taken together, these results suggest that, following a prophylaxis, twice daily use of the SnF_2_ toothpaste resulted in a clinically meaningful early and sustained decrease in gingival bleeding [13–15].

Few other trials have looked at these early timepoints; however, those that have, albeit with procedural differences, concur with the results shown here. For instance, a recent study of a variety of SnF_2_ toothpaste formulations used over 12 weeks had an interim assessment of 2 weeks and also found a significant reduction in number of bleeding sites and gingival index scores at this timepoint. Of note though, this study did not have participants undergo a prophylaxis prior to study toothpaste use [24].

To separate the mechanical effects of toothbrushing and the SnF_2_ toothpaste, another study had participants use a slurry containing either 0.45% w/w SnF_2_ or a control toothpaste without any active anti-gingivitis ingredients [29]. Following prophylaxis, gingival bleeding and gingivitis scores were significantly higher with the control toothpaste after 3 weeks use, showing that, independent of brushing, SnF_2_ is effective for maintenance of gingival health.

While early achievement of low bleeding scores following prophylaxis may be achievable with a SnF_2_ toothpaste, resolution of gingival inflammation may take longer. In this study, for the MGI scores, reduction from baseline for both study products and the difference between them were lower compared to those of other variables. This was also seen in the previous 12 week study, where MGI similarly showed only a small, though statistically significant, difference at Week 4 (5.4%) [14].

This current study also looked at plaque indices as gingivitis develops when plaque elicits a local inflammatory response in the gingivae at the site of its accumulation [27, 30]. It is well established that gingivitis can be prevented and resolved through effective plaque control [4, 30]. SnF_2_ has been shown to exert its plaque control effects via control of microbiome metabolism, which can limit occurrence of inflammatory products that can cause gingival inflammation, such as endotoxins [31, 32]. Here, dental prophylaxis followed by twice daily brushing resulted in reduction of plaque by 16.0% at Week 2 and 15.1% at Week 3. Toothbrushing alone may not reach all parts of the tooth, therefore interproximal plaque scores were of interest as well and a difference was found between the two groups, favouring the SnF_2_ toothpaste (14.6%/14.1% by Weeks 2/3). The studies of this toothpaste at later timepoints (12 and 24 weeks), found similar, sustained differences [13, 15]. This again suggests, as discussed above, that SnF_2_ has a reach beyond that of mechanical toothbrushing.

To assess whether differences in plaque control between SnF_2_ formulations and controls were due to the prophylaxis step, a previous study used a ‘split mouth’ design and had participants brushing with a control toothpaste for 3 weeks while using either a SnF_2_ or control mouthrinse. This study found significant differences in plaque levels between groups after 1 and 3 weeks on both the side of the mouth that had undergone prophylaxis and the non-professionally cleaned side [28]. This indicates that SnF_2_ formulations can be useful for both plaque reduction when a prophylaxis isn’t carried out, and inhibition of plaque formation when such a prophylaxis is administered, as found in this current study.

One limitation of this study was that the BI, MGI and TPI scoring systems depend on examiner judgement. While repeatability was deemed ‘excellent,’ further studies could include less subjective analysis of indices of gingival inflammation such as measures of cytokines and galectins in saliva, serum or tissue. Such studies that have been carried out indicate a relationship between clinically measurable inflammatory factors and periodontitis [33–35]. Both study toothpastes were generally well tolerated with few adverse events reported, all mild, that had resolved by the end of the study.

## Conclusions

In conclusion, this investigation supports the clinical efficacy and tolerability of an anhydrous 0.454% w/w SnF_2_ toothpaste in improving gingival health and shows that gingivitis and plaque control can be shown from as early as 2 weeks following prophylaxis. Although the differences in the gingivitis indices were significantly greater for the 0.454% w/w SnF_2_ toothpaste compared to control, these differences in MGI may not necessarily be clinically relevant at the early timepoints.

## Data Availability

The datasets used and/or analysed during the current study are available from the corresponding author on reasonable request.
